# Pre-harvest spray of GABA and spermine delays postharvest senescence and alleviates chilling injury of gerbera cut flowers during cold storage

**DOI:** 10.1038/s41598-021-93377-4

**Published:** 2021-07-08

**Authors:** Meisam Mohammadi, Mitra Aelaei, Mehdi Saidi

**Affiliations:** 1 Department of Horticulture, Faculty of Agriculture, University of Zanjan, Zanjan, 45371-38791 Iran; 2 Department of Horticulture, Faculty of Agriculture, Ilam University, Ilam, 69315-516 Iran

**Keywords:** Plant physiology, Plant stress responses, Plant sciences, Plant signalling

## Abstract

Short vase life, capitulum wilting, neck bending, and postharvest chilling injury (CI) are major disorders have negative impact on quality and marketing of gerbera cut flowers. Low storage temperatures prolonging the vase life, but on the other hand leads serious CI which decreases the quality and consumer preferences. Spermine (SPER) and γ-aminobutyric acid (GABA) were identified as anti-aging factors delay the senescence and elevate the chilling tolerance in many species. Greenhouse-grown gerbera cv. ‘Stanza’ sprayed with 2 mM SPER and 1 mM GABA twice (2 T) or thrice (3 T). Cut flowers were stored at 1.5 °C and 8 °C postharvest to study the effects of GABA and SPER on senescence and CI. Vase life, CI and quality of cut flowers were improved by GABA and SPER treatments. No CI was observed in GABA-treated flowers at 1.5 °C; while, flowers sprayed with water showed severe CI. GABA treatments efficiently prolonged the vase life for 6–7 days more than the control (15 days). GABA and SPER increased the fresh weight, solution uptake, protein and proline contents, catalase, peroxidase, and superoxide dismutase activities, while decreased the electrolyte leakage, H_2_O_2_, and malondialdehyde contents, polyphenol oxidase, lipoxygenase, and phospholipase D activities. GABA and SPER significantly prolonged the vase life and prevented degradation of proteins and chilling damage and increased capacity of detoxifying and scavenging of H_2_O_2_ and reactive oxygen species (ROS), led to alleviate the negative consequences of the senescence and CI.

## Introduction

Gerbera (*Gerbera jamesonii*), a plant with tropical origin belonging to the Asteraceae family is one of the top 10 most beautiful cut flowers in the world due to its colorful shiny ray florets, their variable shapes and colors, high flower-stem yield and short harvest intervals^1^. However, they often suffer from a short vase life of 5–8 days at room temperatures after harvest^2,3^. The demand for high quality freshly cut flowers has made the development of improved methods of preservation necessary. To make cut flowers last longer and improve their quality and their vase life, many chemical and non-chemical compounds are used, alongside low storage temperature. Among various dynamics to consider when storing cut flowers, temperature is a particularly critical factor. Low-temperature storage is also the most important handling procedure for cut flowers^4^. Cold storage is considered to be an effective approach for improving vase life and preserving quality of cut flowers and other horticultural crops^5^. Gerbera “Savana Red” cut flowers lasted longer at 5 °C compared to 10 or 15 °C^6^. Heat triggers onset of aging and increases respiration rate and ethylene production, while low storage temperatures close to freezing point decelerate respiration-related processes and evapotranspiration, lowering consumption of carbohydrates and other resources of plant cells and delays the senescence. In addition, lower temperatures can reduce the growth of bacteria and fungi, ethylene production, and water loss^5^. However, tissues and organs often damage during storage and handling at low temperatures^7,8^. The problem is profound for tropical ornamental species. Storing tropical cut flowers such as gerbera in vase solutions under cold storage (0–5 °C) or during handling in cargo is always challenging and may induce chilling damage. Consequently, chilling injured cut flowers show wilting, necrosis, and browning of colored bracts and petals which substantially decrease their quality and consumer acceptance^7,9,10^.

Among many compounds use as important protective and preservative factors in cut flowers industry, GABA and major polyamines (PAs), putrescine, spermidine, and spermine, showed positive effects on preserving the quality under abiotic stresses^11–13^. GABA and SPER have been considered as effective anti-aging and anti-chilling agents delay the senescence and elevate the chilling tolerance in many species. Pre- and postharvest GABA treatments alleviated CI and maintained the quality of horticultural crops including peach, banana, and anthurium under cold storage^8–10^.

PAs are poly-cationic hydrocarbons with a low molecular weight carry a linear carbon chain with two terminal amino groups. Putrescine (with two amino groups), spermidine (with three amino groups) and SPER (with four amino groups) are among the most well-known PAs with anti-aging properties, which increase the cellular antioxidant capacity and reduce the ethylene production^9,12,13^. Both ethylene and PAs biosynthesis adhere to the substrate S-adenosine methionine (SAM)^14,15^. It is inferred that aging starts following a reduction in arginine decarboxylase activity and consequently a decrease in the production of PAs^15–17^. PAs can be recognized as substrates or modulators in binding pockets of proteins, phenols, nucleic acids, phospholipids, lignin, and organic acids to protect them against ROS and postharvest CI^13,15,18^. PAs showed positive effects on postharvest quality and vase life of cut roses^18^, lisianthus^19^ and anthurium cut flowers^20^ due to their impacts on ROS and antioxidant capacity. Chilling tolerant tissues are known to have more efficient antioxidant systems against ROS than chilling-sensitive ones which can be due to increase of endogenous PAs levels in response to chilling much greater than chilling sensitive ones^17–21^.

GABA, a non-protein amino acid with four carbon atoms, is generally found in most prokaryotes and eukaryotes. GABA provokes signals in response to environmental stresses and causes proline accumulation which maintains cellular structures, and reduces postharvest damages^9,11,22^. Cell conductance, insect defense responses, energy balance, biotic stress response to fungi, and metabolism of nitrogen and carbon are among other functions attributed to GABA^22^. GABA accumulates in plant cells by the GABA-shunt pathway which is regulated by three key enzymes including glutamate decarboxylase (GAD), GABA transaminase (GABA-T), and succinic semialdehyde dehydrogenase (SSADH)^22^. Under normal cellular pH, GAD binds to calcium-calmodulin complex, but under stress and cellular pH changes, the activity of GAD increases and leads to more GABA synthesis in the cells^23^. The production of GABA is consistent with the consumption of H^+^ due to the activity of GAD. Under chilling stress, an increase in the GAD enzyme activity regulates cellular pH and maintains cytosol acidity at a normal level while CI is alleviated^24^. A few scientific reports revealed the positive effects of GABA on cut flowers. Anthurium cut flowers treated with 1 mM GABA showed lower electrolyte leakage (EL), H_2_O_2_, malondialdehyde (MDA), lipoxygenase (LOX), phospholipase D (PLD) activity and higher proline content and antioxidant enzymes activities than non-treated flowers^11,15,25^. Quality and vase life of rose cut flowers^23^ and gerbera cut flowers^11^ were improved by pre- and post-harvest GABA treatment in vase solution at ambient temperatures.

Gerbera cut flowers suffer from quick senescence and a short vase life at room temperature. The vase life and quality of gerbera cut flowers could improve under low storage temperature, however, as a naturally tropical plant, gerbera is sensitive to CI. Despite the fact that PAs and GABA have been implicated in various important developmental and adaptive processes, their effects on chilling damage and senescence of cut gerbera flowers was not clearly investigated under postharvest cold storage condition. We hypothesized that GABA and SPER may delay senescence, retard or prevent neck bending and CI, and improve postharvest quality of cut gerbera flowers. The present study investigates effects of exogenous GABA and SPER pre-harvest treatments on cut gerbera cv. ‘Stanza’ during postharvest storage under cold temperatures at 1.5 °C and 8 °C, towards better understand and uncover hidden mechanisms involved in delaying senescence and chilling damage under two cold storage temperatures at 1.5 °C and 8 °C. The results can be applied by growers, wholesalers and consumers for better postharvest performance and quality improvement during postharvest storage and handling of cut flowers under cold storage.

## Results

### Vase life of gerbera cut flowers

**Figure 1:**
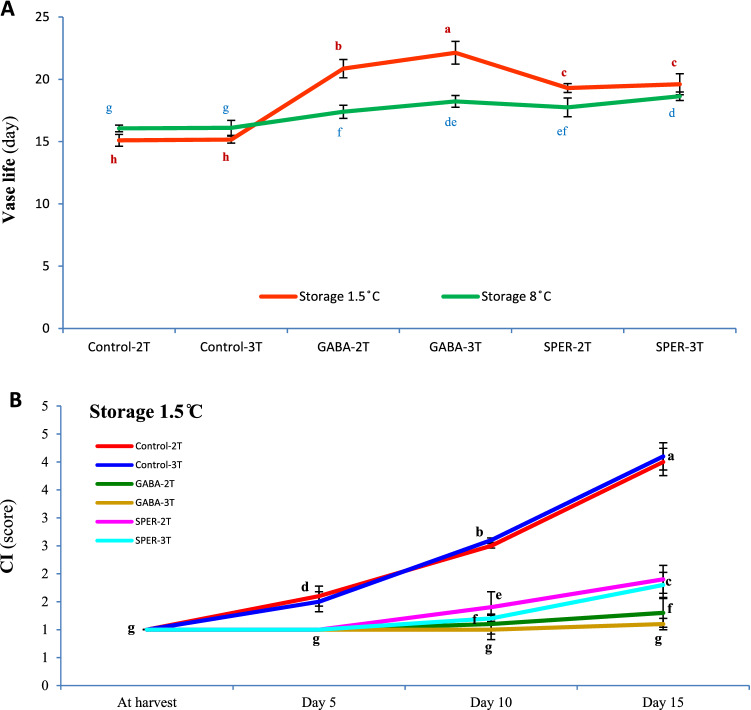
Vase life of the gerbera ‘Stanza’ cut flowers pre-harvest treated with GABA and SPER during cold storage at 1.5 °C and 8 °C (**A**). Chilling injury (CI) in gerbera ‘Stanza’ cut flowers pre-harvest treated with GABA and SPER (**B**). 2 T and 3 T represent number of times the GABA and SPER are sprayed on intact gerbera plants at pre-harvest. Values are mean SE, n = 4. The bars marked with the same letters are not significantly different at p < 0.05.

**Figure 2:**
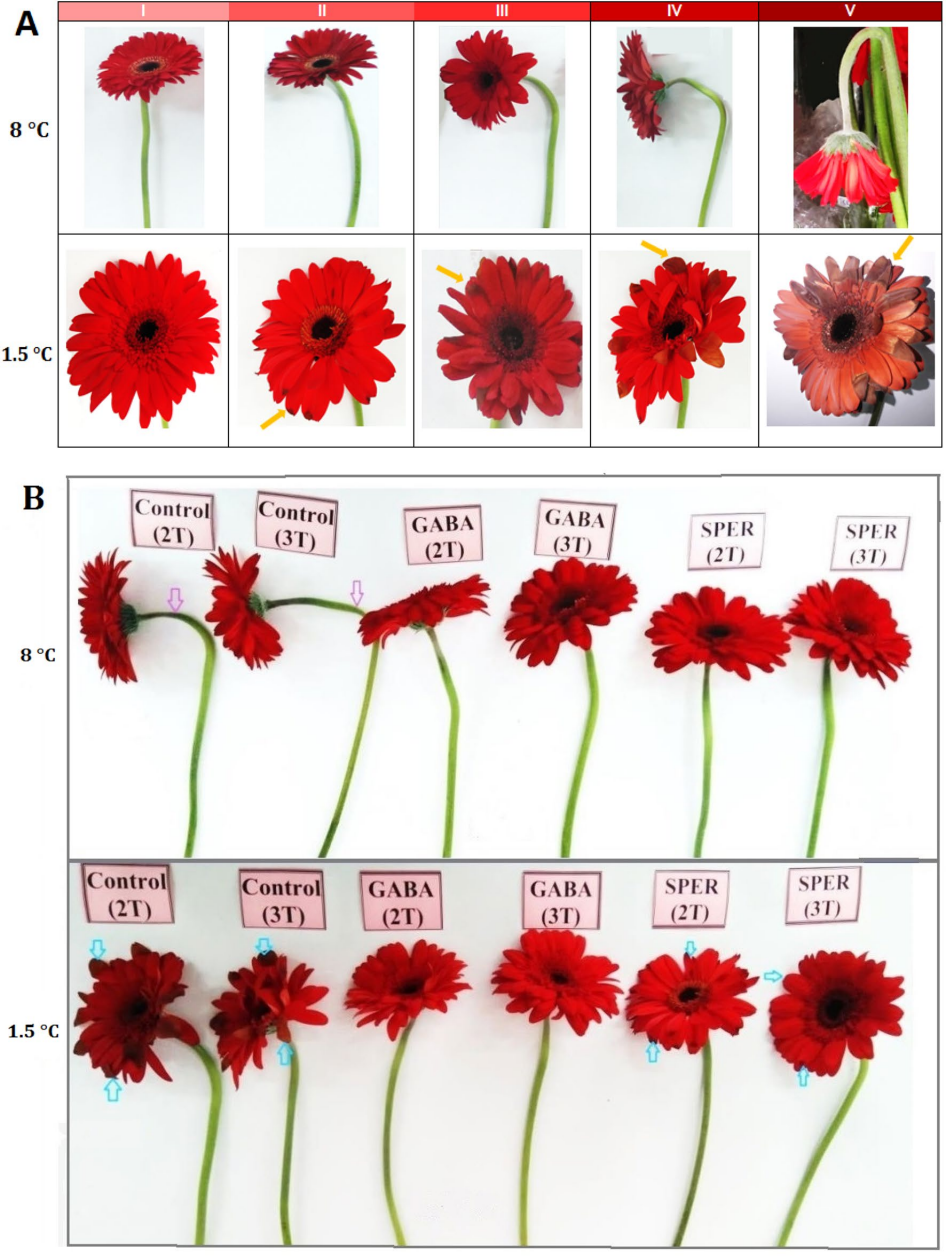
Schematic descriptor of the neck bending (Stage I-V) and chilling injury (CI) in gerbera ‘Stanza’ cut flowers during cold storage at 1.5 °C and 8 °C. I = no CI; II = mild CI (up to 10% chilling of capitulum); III = moderate CI (up to 30% chilling of capitulum); IV = severe CI (up to 50% chilling of capitulum); V = very severe CI (70–100% chilling of capitulum) (**A**). Effects of pre-harvest GABA and SPER treatments on the neck bending and chilling injury of the gerbera ‘Stanza’ cut flowers during cold storage at 1.5 °C and 8 °C, 15 days postharvest (dph) (**B**).

The vase life of cut flowers ranged between 15 and 22 days (Fig. 1A). The longest vase life was observed in cut flowers were kept at 1.5 °C. According to the experimental observation, water-sprayed (Control) cut flowers maintain their quality and freshness to 15 days postharvest (dph), whereas, GABA treatments efficiently prolonged the vase life for more than six (2 T) to seven (3 T) days at 1.5 °C, without any visible CI. Cut flowers pre-harvest sprayed with GABA lasted longer for 22 days (GABA-3 T) and 21 days (GABA-2 T) relative to SPER-treated flowers with an average vase life of 19 days (SPER-2 T and SPER-3 T) at 1.5 °C (Fig. 1A). However, at 8 °C the longest vase life was seen in SPER-3 T (18.6 days), GABA-3 T (18.2 days), SPER-2 T (17.7 days) and GABA-2 T (17.4 days) respectively (Fig. 1A). A slighter neck bending was observed at 1.5 °C compared to 8 °C. Indeed, cut flowers sprayed with deionized water (Control) showed visible neck bending over time at 8 °C (Fig. 2B). Control water-sprayed cut flowers showed sever neck bending compared to GABA- and SPER-treated flowers (Fig. 2B).

### Visible symptoms of CI

No visible CI was observed at 8 °C, whereas, cut flowers sprayed with deionized water (Control) showed visible severe CI overtime at 1.5 °C (Fig. 1B). No significant CI was observed in GABA-treated flowers at 1.5 °C. Cut flowers sprayed with SPER, however, showed mild injuries 6–15 dph (Figs. 1B, 2B; Supplementary Fig. S1).

### Fresh weight (FW) and vase solution uptake (VSU)

**Figure 3:**
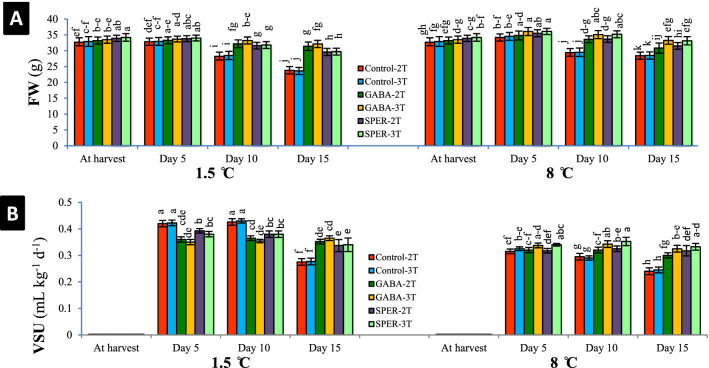
Effect of pre-harvest GABA and SPER spray on the fresh weigh (FW**)** (**A**) and vase solution uptake (VSU) (**B**) of gerbera ‘Stanza’ cut flowers during cold storage at 1.5 °C and 8 °C. 2 T and 3 T represent number of times the GABA and SPER are sprayed on intact gerbera plants at pre-harvest. Values are mean SE, n = 4. The bars marked with the same letters are not significantly different at p < 0.05.

During cold storage at 1.5 °C, fresh weight of cut flowers showed an upward consistent pattern in all treatments to 5 dph; however, a slight decrease was observed 10 and 15 dph. GABA- and SPER-treated flowers demonstrated higher FW than water-sprayed control cut flowers 15 dph. At 8 °C, the FW revealed a significant decrease 10 and 15 dph, after a rise during 5 dph (Fig. 3A, Supplementary Fig. S2A). VSU showed gradual reduction over time. On average, during the period of 15 days, the smallest amount of absorbed solution was for water-sprayed controls and the largest for the GABA- and SPER-treated cut flowers stored at 1.5 °C. At 8 °C, the largest was observed in SPER-3 T and GABA-3 T respectively (Fig. 3B, Supplementary Fig. S2B).

### Membrane integrity related to EL and MDA contents

**Figure 4:**
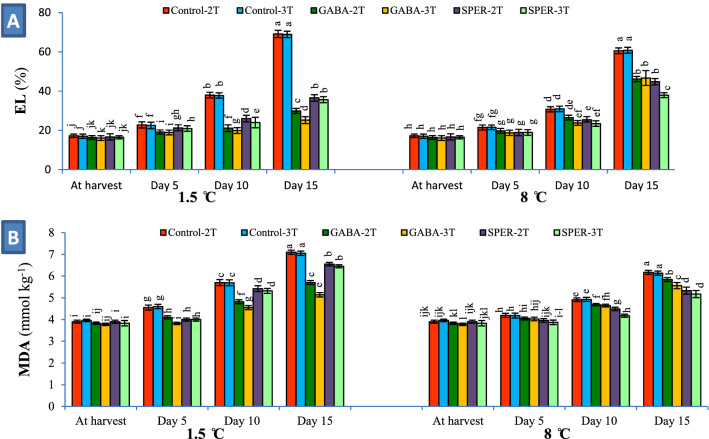
Effect of pre-harvest GABA and SPER spray on the electrolyte leakage (EL) (**A**) and malondialdehyde (MDA) content (**B**) in gerbera ‘Stanza’ cut flowers during cold storage at 1.5 °C and 8 °C. 2 T and 3 T represent number of times the GABA and SPER are sprayed on intact gerbera plants at pre-harvest. Values are mean SE, n = 4. The bars marked with the same letters are not significantly different at p < 0.05.

Measuring the EL and MDA contents to analyze the status of membrane integrity demonstrated that the cell membranes were degraded over the storage time at both 1.5 °C and 8 °C. EL values in cut flowers increased during storage (Fig. 4A). Level of EL in cut flowers sprayed with deionized water was significantly higher than that of GABA- and SPER-treated cut flowers 15 dph (Control-2 T = 69.12%, Control-3 T = 68.90% at 1.5 °C; and Control-2 T = 60.55%, Control-3 T = 60.80% at 8 °C). The relative increase of EL in petals of flowers sprayed with GABA and SPER was lower (GABA-3 T = 25.21%, GABA-2 T = 29.89%, SPER-3 T = 35.62%, SPER-2 T = 36.55% at 1.5 °C; and SPER-3 T = 37.92%, SPER-2 T = 44.80%, GABA-2 T = 46.15%, GABA-3 T = 46.67% at 8 °C, respectively), represented a better membrane integrity (Fig. 4A, Supplementary Fig. S3A). Content of MDA in cut flowers sprayed with deionized water was higher than that of GABA- and SPER-treated samples (Control-2 T = 7.11 mmol kg^−1^, Control-3 T = 7.05 mmol kg^−1^ at 1.5 °C; and Control-2 T = 6.17 mmol kg^−1^, Control-3 T = 6.13 mmol kg^−1^ at 8 °C) indicating that GABA and SPER could protect the plasma membrane from ROS attacks (Fig. 4B, Supplementary Fig. S3B).

### Total protein, proline and H_2_O_2_ contents

**Figure 5:**
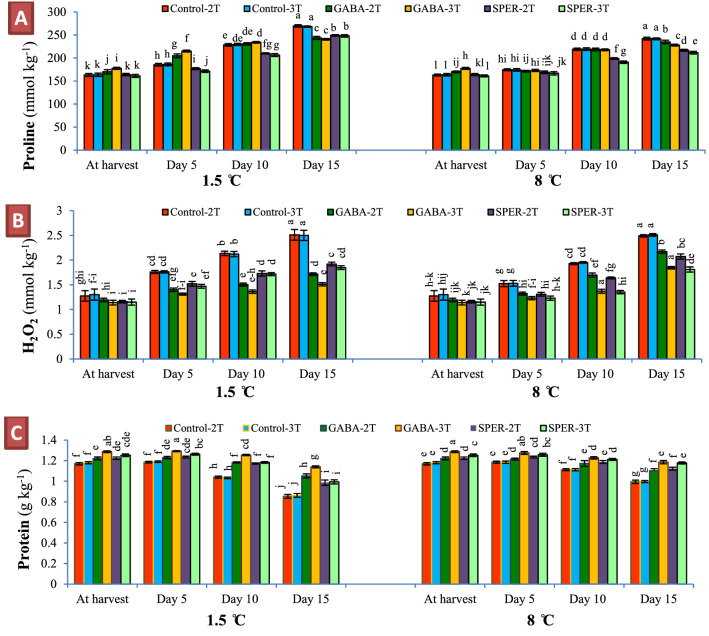
Free proline (**A**), hydrogen peroxide (H_2_O_2_) (**B**) and total protein (**C**) contents in gerbera ‘Stanza’ cut flowers pre-harvest treated with GABA and SPER, during cold storage at 1.5 °C and 8 °C. 2 T and 3 T represent number of times the GABA and SPER are sprayed on intact gerbera plants at pre-harvest. Values are mean SE, n = 4. The bars marked with the same letters are not significantly different at p < 0.05.

The free proline content increased in petal cells with the progressing senescence over time at 1.5 °C and 8 °C. GABA and SPER treatments decreased the proline content in petals compared to the controls 15 dph. The proline content in GABA-3 T and GABA-2 T was 240.8 and 243.5 mmol kg^−1^ respectively at 1.5 °C. However, SPER-3 T (211.7 mmol kg^−1^) and SPER-2 T (216.7 mmol kg^−1^) showed the lowest proline content at 8 °C. Pre-harvest water-sprayed controls showed the highest proline content 15 dph (Fig. 5A, Supplementary Fig. S4A). The H_2_O_2_ content increased over time to 15 dph in all treatments. However, H_2_O_2_ content drastically increased on day 5 to day 15 in water-sprayed flowers compared to pre-harvest GABA- and SPER-treated flowers. The smallest changes in H_2_O_2_ content were in cut flowers sprayed with GABA and SPER. GABA-treated flowers showed the lowest H_2_O_2_ content at 1.5 °C (GABA-3 T = 1.51 mmol kg^−1^, GABA-2 T = 1.71 mmol kg^−1^) while at 8 °C, SPER-3 T (1.81 mmol kg^−1^) and GABA-3 T (1.84 mmol kg^−1^) treatments demonstrated the lowest H_2_O_2_ content respectively (Fig. 5B, Supplementary Fig. S4B). Protein content in petals decreased with the progressing senescence over time at both 1.5 °C and 8 °C. However, on 15 dph, pre-harvest GABA- and SPER-treated flowers showed higher total protein content than pre-harvest water-sprayed controls, revealing less protein degradation. On day 15, the largest amounts of proteins were detected in cut flowers sprayed with GABA-3 T and kept at 1.5 °C (1.14 g kg^−1^). At 8 °C, the largest amounts of proteins were detected in cut flowers treated with GABA-3 T (1.18 g kg^−1^) and SPER-3 T (1.17 g kg^−1^) (Fig. 5C, Supplementary Fig. S4C).

### Antioxidant enzyme activity

**Figure 6:**
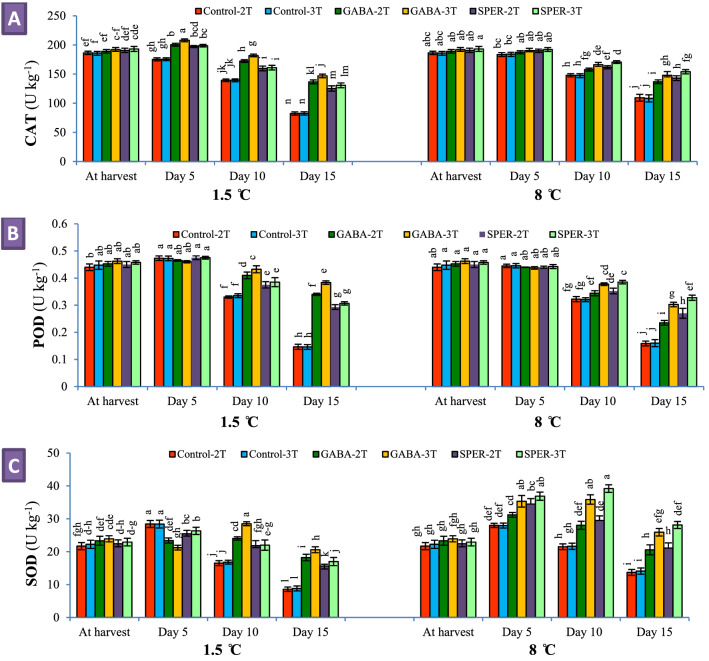
Catalase(CAT)(**A**), Peroxidase (POD) (**B**) and Superoxide dismutase (SOD) (**C**) activities in gerbera ‘Stanza’ cut flowers pre-harvest treated with GABA and SPER, during cold storage at 1.5 °C and 8 °C. 2 T and 3 T represent number of times the GABA and SPER are sprayed on intact gerbera plants at pre-harvest. Values are mean SE, n = 4. The bars marked with the same letters are not significantly different at p < 0.05.

Activity of ROS-scavenging system in gerbera cut flowers significantly affected by GABA and SPER treatments during cold storage. The CAT activity dropped drastically in water-sprayed controls with the progressing senescence over time at both 1.5 °C and 8 °C. On day 15, the lowest CAT activity was observed in cut flowers sprayed with water (Control-2 T and Control-3 T). In GABA- and SPER-treated flowers, the CAT activity increased slightly approx. 1.1 times during day 0 and 5 and then decreased during the following 10 days, but remained at a higher level than the controls. Storage stimulated the CAT activity in treated cut flowers, at the start of the vase life. The highest CAT activity was observed in GABA-3 T (147 U kg^−1^) and GABA-2 T (137 U kg^−1^) treated flowers stored at 1.5 °C. The highest CAT activity was observed in SPER-3 T (154 U kg^−1^) and GABA-3 T (148 (U kg^−1^) at 8 °C respectively (Fig. 6A, Supplementary Fig. S5A).

Analyses done immediately after harvest and on day 5 showed no significant changes in POD activity at 1.5 °C and 8 °C. POD activity then dropped drastically (approx. up to 3 times) in water-sprayed flowers over time. In GABA- and SPER-treated flowers, the POD activity decreased more slightly approx. 1.2–1.5 times at 1.5 °C and 1.3–1.8 times at 8 °C during day 5 and 15 and remained at a higher level than the controls (Fig. 6B, Supplementary Fig. S5B).

SOD activity increased approx. 1.3 times during day 0 and 5 in water-sprayed controls at both 1.5 °C and 8 °C. Its activity then dropped drastically (approximately 2–3 times at 8 °C and 1.5 °C respectively) with the progressing senescence over time. SOD activity in GABA- and SPER-treated flowers revealed a significant decrease 15 dph, after an initial rise on day 5 and 10 (Fig. 6C, Supplementary Fig. S5C). Cut flowers sprayed with GABA-3 T showed the maximum POD (0.38 U kg^−1^) and SOD activity at 15 dph (20.60 U kg^−1^). The relative reduction in CAT, POD and SOD activity in the GABA- and SPER-sprayed flowers was lower compared to the controls. Cut flowers sprayed with SPER-3 T and GABA-3 T demonstrated the highest POD activity (0.32 and 0.30 U kg^−1^ respectively) and SOD activity (28.31 and 25.94 U kg^−1^ respectively) compared to the controls at 8 °C (Fig. 6, Supplementary Fig. S5).

### PPO, LOX and PLD activity

**Figure 7:**
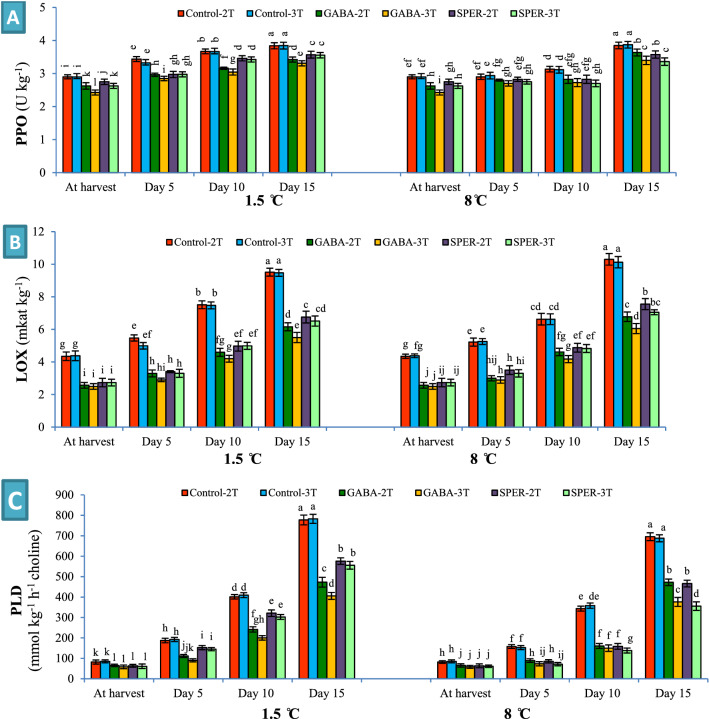
Polyphenol oxidase (PPO) (**A**), lipoxygenase (LOX) (**B**) and phospholipase D (PLD) (**C**) activities in gerbera ‘Stanza’ cut flowers pre-harvest treated with GABA and SPER, during cold storage at 1.5 °C and 8 °C. 2 T and 3 T represent number of times the GABA and SPER are sprayed on intact gerbera plants at pre-harvest. Values are mean SE, n = 4. The bars marked with the same letters are not significantly different at p < 0.05.

PPO, LOX and PLD activities of cut flowers increased over time during storage at 1.5 °C and 8 °C, but the trend was slower in all GABA- and SPER-treated samples compared to the controls. LOX and PLD activities increased drastically in water-sprayed controls with the progressing senescence at 1.5 °C and 8 °C. Cut flowers sprayed with GABA-3 T had the lowest PPO (3.3 U kg^−1^), LOX (5.5 mkat kg^−1^), and PLD (405 mmol kg^-1^ h^−1^ choline) activities. At 8 °C, SPER-3 T and GABA-3 T showed the lowest PPO activity (3.3 U kg^−1^). On day 15 the lowest LOX activity was in GABA-3 T at 8 °C (6.1 mkat kg^−1^). SPER-3 T (355 mmol kg^−1^ h^−1^ choline) and GABA-3 T (376 mmol kg^−1^ h^−1^ choline) showed the lowest PLD activity (Fig. 7, Supplementary Fig. S6).

## Discussion

Gerbera along with other popular flowers such as roses, lilies, and anthurium, is one of the valuable cut flowers in considerable demand for both domestic and export markets^11^. Gerbera is widely used because of high decorative value of its colorful flowers. However, gerbera cut flowers suffer from relatively short vase life of 5–8 days at room temperature^2^ and about two weeks at cold storage condition without treatment (this study). After harvest, cut stems should be rapidly cooled, down to about 1 °C. Under this temperature, cut flowers can be stored for several more considerable days, however, as a naturally tropical plant; gerbera is sensitive to CI. Short vase life at room temperature and postharvest CI under cold storage have negative impact on quality and marketing of gerbera cut flowers. Therefore, this study was conducted to sustain gerbera cut flowers under cold storage for a longer period with minimal injuries from chilling, using potentially anti-aging and anti-chilling agents such as GABA and SPER. GABA and SPER treatments had significant impacts on vase life quality and CI of gerbera cut flowers under cold storage. While the storage temperature was decreased from 8 to 1.5 °C, the chilling damage rose drastically in control cut flowers which led to the decline of the vase life. GABA enhanced the antioxidant capacity and improved scavenging of H_2_O_2_ and ROS, decreased the activity of PPO, LOX and PLD enzymes, and MDA and EL contents in treated cut flowers, hence prolonged the vase life 7 more days without any visible CI. Similarly, antioxidant capacity related to CAT, POD, and SOD were increased in SPER-treated flowers. Trend of increasing PPO, LOX and PLD activities in treated flowers was slower. SPER decreased the H_2_O_2,_ EL and MDA contents of cut flowers compared to the controls. The findings reveal that SPER reduce the damage of ROS, thus maintaining the normal function of cells during cold storage, which delays the aging process and chilling^14,16,25^. Recent studies indicate that SPER functions differently during biotic and abiotic stresses and induces phytohormone signaling, gene networks and both non-enzymatic and enzymatic antioxidant pathways^12,25^. Moreover, PAs could bind to negatively-charged molecules such as nucleic acids, phospholipids and proteins, thereby protecting them from degradation and modification^13–15^.

Proline accumulation affected by GABA and SPER treatments during cold storage led to reduce chilling damage in the present study. It has been suggested that proline accumulates within cells during cold stress, reduces cold damage and extends postharvest vase life^8,11,26^. The free proline content increased in petal cells with the progressing senescence over time. The internal proline content was higher in water-treated controls than those treated with GABA and SPER 15 dph, which could be due to the breakdown of proteins in the respiratory process caused by uplifted stress and aging of flowers during postharvest storage. The GABA and SPER treatments reduced cell stress and delayed aging by enhancing antioxidant capacity, maintaining cell wall strength, and probably decreasing respiration, which decelerates protein degradation by reducing ROS activity in cut flower’s cells and decreased proline content in the GABA and SPER treatments compared to controls. In the present study, treatments with lower protein content showed higher proline content too, which could be due to protein degradation. GABA and SPER treatments brought about higher protein content at harvest time than the controls, which could be due to the protective effect by lowering the pre-harvest stresses and providing optimum conditions for photosynthesis and storage of metabolites in the cells before harvest. The decrease in proline and total protein contents of different cultivars of gerbera treated with SPER^11^ and nitroprusside^31^ were reported. Reduced chilling stress followed by GABA treatment in anthurium cut flowers^10,26^ and banana fruits^9^ was attributed to proline accumulation in cells. Radhakrishnan et al*.* (2014) reported that exogenous application of SPER increased plant growth by increasing total protein^27^. Shelp et al. (2012) reported the role of GABA signaling in nitrogen accumulation, as a factor contributing to higher total protein content of gerbera cut flowers by GABA treatments^28^. A large body of data suggests a positive correlation between proline accumulation and plant stress due to acting as an osmolyte, cell membrane stabilizer, and a metal chelator, an antioxidative and signaling molecule^29^.

PAs accumulate in plant cells under cold stress conditions and delay the aging of horticultural crops due to decrease in polyamine oxidase activity and ethylene production and an increase in protease and ribonuclease activities^12,13^. In addition, PAs alleviate the damage of ROS and induce cold stress resistance by boosting the antioxidant capacity and binding to the phospholipid portion of cell membranes^15,21^. It has been reported that SPER inside the cell can be converted into 4-aminobutanal, then to pyrroline, and finally to GABA which alleviates postharvest stress and cell damage, and enhances postharvest life of horticultural crops^16^. The increase of internal GABA and proline content (without decreasing the protein content) after GABA treatment may explain better performance of GABA than SPER to decrease the chilling damage at 1.5 °C cold storage. Boosting the activity of antioxidant enzymes followed by SPER treatments may also have a major impact on maintaining the quality of gerbera cut flowers and decreasing the CI in the present study. Prolonged vase life and postharvest quality of gerbera^11,30^ and rose cut flowers^18^ have been previously reported followed by pre- and postharvest polyamine treatments. Longer vase life of gerbera in cold storage has been related to less respiration, less bacterial growth, and vascular obstruction, and more water uptake^3,5^. Chilling damage of anthurium cut flowers was reduced by GABA treatment due to increase in antioxidant capacity and proline content, and decrease in EL, H_2_O_2_ and MDA content^10^.

Changes in FW and VSU during storage show downward trends due to vascular blockage and insufficient water absorption, neck bending and the prevention of easy mobilization of water to the flower disc, aging process, and cellular turgor loss^10,11,31^. In addition, under cold stress, cell membrane lipids are transformed from liquid-crystalline to solid-gel state, which increases the permeability of the cell membrane and increases the ions leakage. Under such conditions, cellular turgor, VSU ability, and subsequent FW of cut flowers decrease and respiration and postharvest losses increase^10,11,26^. Our results showed that samples with higher VSU had higher FW and lower CI during storage. The reduction of CI followed by GABA and SPER treatments was related to higher uptake of the vase solution. A lower neck bending in GABA and SPER treated cut flowers may be due to higher vase solution uptake, higher FW, preserved more cellular turgor and less vascular blockage^11,32^. Perik et al*.* (2012) reported bending of gerbera cut flowers during postharvest due to net water loss from the stem, particularly in the area of bending, and to low mechanical in the upper part of the stems, which lack a sclerenchyma cylinder^32^. The PAs contributes to adapting to low temperature by maintaining the fluidity of the membrane and increasing unsaturated lipids of the cell membrane, which is an important factor to maintaining cellular turgor and water absorption ability of cut flowers during cold storage by SPER treatments^29^.

ROS production during different stresses can cause lipid peroxidation of membranes, which enriches the MDA content as the end product and indicates the destruction and permeability of cell membranes^33^. CI first occurs in cell membranes with a change in the phospholipid fatty acid composition^10^. PLD is the main enzyme in the hydrolysis of membrane phospholipids. In addition, LOX activity elevates peroxidation of cell membrane lipids and changes membrane fluidity and has a direct effect on cell membrane integrity and permeability. The activity of these enzymes initiates cell membrane degradation during aging and postharvest chilling stress^9,10^. Amino compounds in cell membranes decrease their postharvest stress and improve their stability by binding to negative ions of phospholipids or anionic segments on the membranes, thereby reducing the synthesis of ROS, EL, and MDA content during postharvest storage^29^. In addition, the binding of PAs to pectic complexes of membranes reduces the access of cell wall degenerating enzymes such as polygalacturonase, exo-polygalacturonase, and methylesterase to pectin compounds, and mitigates damages to cell membranes by enhancing antioxidant capacity and reducing ROS^16^. GABA and SPER treatments inhibited the activity of the LOX and PLD enzymes in gerbera cut flowers at both storage temperatures, which preserved the structure of cell membranes and decreased the MDA content and EL leading to CI alleviation and vase life extension.

Under normal storage temperatures SOD, CAT, POD, glutathione reductase and ascorbate peroxidase keep superoxide radicals (O_2_^−^) and H_2_O_2_ at low levels. However, when storage temperatures drop below a safe point, cold stress is induced^4,11^. In *Lantana camara* and *Heliotropium arborescens* the CI symptoms were associated with significant increases in ROS production after storage at 2 °C, 4 °C, 6 °C, or 8 °C^35^. In gerbera, SPER alleviates oxidative damage through the stimulation of ROS-scavenging enzymes, leading to an antioxidant response^13,15,25^. The relative reduction in CAT, POD and SOD activities in the GABA- and SPER-sprayed flowers was lower compared to the controls. Although, the enzymes activities reduced over time compared to day 0 (at harvest), but remained at higher level than the controls on day 15. One of the phenomena occurring during senescence of cut flowers is accumulation of ROS and activation of the enzymatic antioxidant defense mechanism. Induction of the aging process consequently increases the amount of ROS and triggers the antioxidant system^11,34–36^. PAs maintain the antioxidant capacity of cut flowers by lowering postharvest stress and ROS production^11,16^. The activity of antioxidant enzymes was enhanced in banana fruits by GABA treatment, which was attributed to the signaling role of GABA and preserving antioxidant compounds by reducing ROS synthesis^9^. The higher activity of antioxidant enzymes in anthurium cut flowers treated with GABA and salicylic acid were attributed to the reduction of the postharvest stress and respiration of the treated flowers and decrease in the consumption of antioxidant compounds to scavenge ROS^26^. In another study, the treatment of anthurium cut flowers with GA_3_ and SPER contributed to maintaining CAT and POD activity during storage^20^.

**Figure 8:**
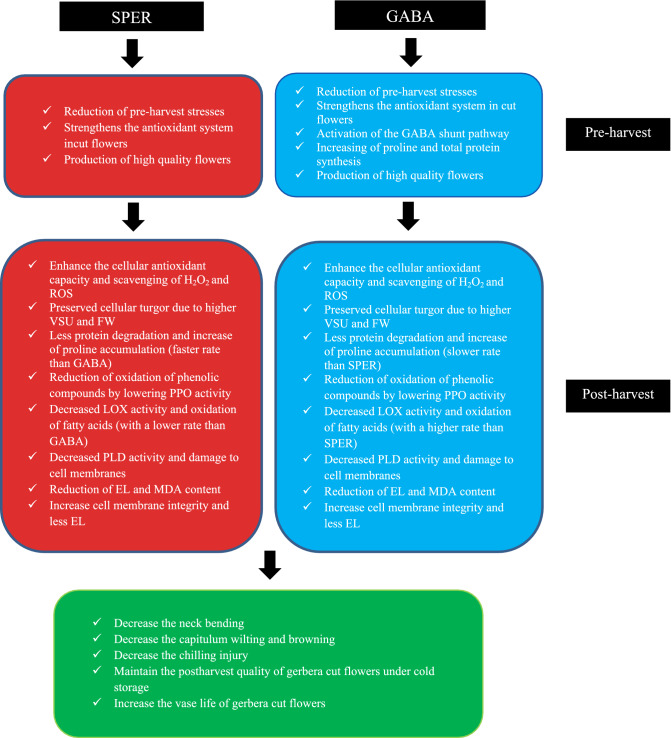
A proposed model for GABA and SPER-mediated chilling tolerance in gerbera cut flower during postharvest cold storage at 1.5 °C and 8 °C. (*ROS* reactive oxygen species, *VSU* vase solution uptake, *FW* fresh weigh, *PPO* polyphenol oxidase, *LOX* lipoxygenase, *PLD* phospholipase D, *EL* electrolyte leakage, *MDA *malondialdehyde).

During cold stress, the production of ROS leads to oxidative stress responses and consecutive damages to proteins, DNA, and lipids. Lower EL, H_2_O_2_ and MDA content in treated flowers led to higher capacity of scavenging of H_2_O_2_ and ROS, and lower stress and subsequently PPO activity was reduced^4^. Internal and external browning of tissues occurs during the oxidation of phenolic compounds due to PPO activity^15^. PPO is responsible for converting phenolic compounds into quinines, which can be converted to ROS under radiation^11,37,38^. PAs inactivate ROS and protect cell membranes from oxidation. While internal PAs rise, EL and petal browning diminish^37^. Lower chilling injury was correlated with lower PPO activity in gerbera ‘Stanza’ cut flowers in the present study. Lower chilling damage, less color retention, and delay in the browning of anthurium cut flowers^10,15^, banana fruits^9^ and peach fruits^8^ was attributed to GABA due to lower PPO activity and higher activity of antioxidant enzymes. According to the results obtained, a schematic model was proposed for GABA and SPER-mediated chilling tolerance in gerbera cut flower during postharvest cold storage at 8 °C and 1.5 °C (Fig. 8).

## Conclusion

The data indicate the importance of maintaining the optimal cold temperatures during storage and commercial handling procedure of gerbera cut flowers to obtain maximum vase life and quality. However, the long-term cold storage leads serious CI in gerbera cut flowers, which substantially decreases their quality and vase life. Our results indicate that pre-harvest GABA and SPER treatments at a proper concentration has improving effect on the quality and vase life of gerbera cut flowers while diminishing chilling injuries to zero through GABA treatment. Overall, GABA and SPER play important roles in preventing chilling damage and postponing the senescence due to their anti-aging properties and their capability to increase proline content, enhance the cellular antioxidant capacity and scavenging of H_2_O_2_ and ROS, and increase cell membrane integrity and fluidity. The preliminary proposed mechanisms of GABA and SPER to mediate chilling tolerance in gerbera cut flower during postharvest cold storage can serve as a starting point for future in-depth studies, with the aim of implementing effective practical approaches by industry to extend the vase life, prevent neck bending and chilling injury during transportation of tropical cut flowers over long distances. The results not only can be applied in better postharvest performance and quality improvement as well as sustaining gerbera cut flowers under cold storage, but also may improve cold chain logistics in the floral industry. In future work, we aim to focus on the series of molecular mechanisms and gene networks underlying physiological protective effects of GABA during cold storage and explore how they work in chilling process.

## Materials and methods

### Plant material, treatments and experimental design

To test the effect of pre-harvest SPER and GABA treatments on vase life and quality of gerbera cut flowers, a commercial cultivar ‘Stanza’ were obtained from a legalized local commercial greenhouse in Pakdasht, Tehran (latitude: 35°28′54′′N, longitude: 51°40′49′′E). Plants with similar size and development stage were chosen with the permission. All protocols were complied with relevant institutional, national, and international guidelines and legislation. While growing, the plants were sprayed with either 2 mM SPER or 1 mM GABA. The SPER and GABA concentrations were selected based on our previous study^10^ and preliminary experiments (Supplementary Fig. S7, S8, S9). The concentrations of the treatments were chosen within the limits of the lowest effective dosage with the most impacts considering economic issues. Plants sprayed with distilled water constituted the control group. Plants were either sprayed twice (2 T) until the emergence of more than 50% of flowering shoots and 5 days later, or thrice (3 T) until the emergence of more than 50% of flowering shoots and 5 days and 10 days later. At commercial maturity stage (in which 3–4 whorls in the floral head showed mature statement and uniformity in size and maturity), a total of 1728 healthy flowers uniform in size, shape and maturity were cut using a sharp sterile knife in the early morning, transferred immediately into sterile buckets filled with deionized distilled water. The buckets were covered with transparent plastic films to minimize water evaporation and transported to the postharvest laboratory where the flower stem ends were re-cut at a length of 45 cm under water to remove air embolism and prevent vascular blockage. Re-cut flowers were placed in glass bottles containing 500 mL of 0.001 g L^−1^ 8-hydroxyquinoline citrate and 1% w/v sucrose as the vase solution and were stored either at 1.5 °C or 8 °C. Cut flowers together with glass bottles were randomly divided into six groups in four replicates (144 cut flowers for each Control-2 T, Control-3 T, GABA-2 T, GABA-3 T, SPER-2 T and SPER-3 T). The experiment was carried out in controlled thermal and light conditions and important physiological and biochemical attributes of cut flowers were investigated at 1.5 °C and 8 °C cold storage temperature in a standard chamber containing 85 ± 5% RH until day 15 (at harvest time and 5th, 10th and 15th days postharvest storage). At 15 dph, the cut flowers were removed from the chamber and were kept at 21 ± 1 C, 65 ± 5% RH and 12 h of lightness at 20 μmol m^−2^ s^−1^ until the end of the vase life. The 15 dph was considered as the end vase life of control cut flowers at 1.5 °C.

### Vase life, CI index, fresh weight (FW) and vase solution uptake (VSU)

The values of > 60% petal wilting and > 90° neck bending (by a protractor) or breaking were considered as the end of vase life. To assay the postharvest vase life and quality of gerbera cut flowers, a descriptor was developed based on neck bending and CI (Fig. 2A). The extent of capitulum browning and wilting were scored visually in 12 individual cut flowers from I-V; where I = no CI; II = mild CI (up to 10% chilling of capitulum); III = moderate CI (up to 30% chilling of capitulum); IV = severe CI (up to 50% chilling of capitulum); V = very severe CI (70–100% chilling of capitulum). The CI was calculated as Eq. (1) in according to Promyou et al. (2012)^39^, where N_1_CI, N_2_CI and NT, show the CI score, the number of flowers with a certain CI score and the total number of flowers in each group respectively. The fresh weight (FW) of cut flowers was measured by a digital scale (0.01 g precision) at harvest time and during postharvest storage. The vase solution uptake (VSU) was calculated using Eq. (2) in which St and St_1_ show the weight of the solutions at investigated times (5, 10 and 15 days) and the previous day in kg, respectively^15^.

1 $${\text{CI}} = \sum \frac{{\left( {N1CI \times N2CI} \right)}}{{NT}}$$

2 $${\text{CI}} = {\text{St}}_{{\text{1}}} - {\text{St}}$$

### Membrane integrity

To determine the EL, petals pieces with the same thickness (1 g, n = 4) were cut by hand punching. Pieces were washed by distilled water and put into test tubes containing 10 mL of deionized distilled water, and shaken at 25 °C on a shaker (150 × g) for 4 h. EC_1_ (Electrical Conductivity) was measured using a conductometer. To determine the EC_2_, the petal rings were then autoclaved (120 °C) for 20 min to release all the electrolytes inside the cells. After cooling to room temperature, electroconductivity was measured again to determine the total electrolyte content (EC2)^38^. The EL was expressed as a percentage of its total content in the tissue, according to the formula EL% = (EC1/EC2) × 100.

MDA content of petals was measured with the method described by Hodges et al. (1999)^40^.

### Free proline and H_2_O_2_ contents

To assay the free proline content, petals (0.5 g, n = 4) were homogenized in 5 mL of 3% aqueous 5-sulfosalicylic acid solution. After centrifugation for 20 min at 12,000 × g, supernatant (1 mL) was used to measure free proline content at 520 nm (Scinco, S-3100) as described by Zhang et al. (2010)^41^. The H_2_O_2_ content of petals was measured spectrophotometrically as previously described by Nasr Esfahani and Mostajeran (2011)^42^.

### Total protein and enzymes assay (CAT, POD, SOD and PPO activities)

Total protein content was measured based on the binding of Coomassie Brilliant Blue G-250 to protein according to the Bradford method using the samples were stored in −80 °C^43^. The petal samples (0.5 g, n = 4) were homogenized in a cooled mortar with 50 mL of potassium phosphate buffer (50 mM, pH 7.8) containing 2% (w/v) polyvinylpyrrolidone (PVP) and centrifuged (10,000 × *g* for 15 min at 4 °C). The clear transparent was used to measure the enzyme activity. The enzyme activity was represented as unit kg^-1^ FW (U kg^-1^) for all enzymes. Aliquots of supernatant were used directly to quantify the activity of catalase (CAT) spectrophotometrically at 290 nm based on the method described by Zhang et al*.* (2013)^1,44^. The Superoxide dismutase (SOD) activity was assayed by the method described by Zhang et al. (2013)^44^. One unit of the SOD activity (U) was defined as the amount of enzyme that inhibited the photoreduction of 50% nitro blue tetrazolium (NBT), measured at 560 nm (Scinco, S-3100). Peroxidase (POD) activity was quantified based on the method described by Zhang et al*.* (2013)^44^. A reaction mixture containing 2.77 mL potassium phosphate buffer (50 mM, pH 7.8), 100 μL of 1% H_2_O_2_, 100 μL of 2% guaiacol, and 30 μL of supernatant was used to measure the POD activity using a spectrophotometer (Scinco, S-3100) at 470 nm for 3 min^35,44^. A reaction mixture containing 2.77 mL potassium phosphate buffer (50 mM, pH 7.8), 200 μL of 0.02 M pyrogallol, and 100 μL of supernatant was used to measure the polyphenol oxidase (PPO) activity at 420 nm^44,45^.

### LOX and PLD activities

LOX and PLD activities were quantified according to the method described by Soleimani Aghdam et al*.* (2016a)^26^. To assay LOX activity, petal tissues (1 g) were homogenized in 10 mL of phosphate buffer (100 mM and pH 8.0) containing 2% PVP. The homogenate was centrifuged at 12,000 × *g* for 30 min at 4 °C. The clear transparent was used to assay LOX activity. A reaction mixture containing 2.4 mL phosphate buffer (100 mM, pH 6.8), 0.1 mL sodium linoleic acid solution (10 mM), and 0.5 mL of supernatant was used to quantify the LOX activity in mkat kg^−1^ FW. One unit of the LOX activity was defined as an increase in absorbance at 234 nm of 0.1 per min per milligram of protein under assay conditions. To quantify the PLD activity, petal tissues (1 g) were homogenized in 10 mL of Tris–HCl (50 mM, pH 8.0) containing 200 mM sucrose, 10 mM of KCl, 2% PVP (w/v) and 0.5 mM of PMSF. After centrifuging at 12,000 × *g* for 30 min at 4 °C, the supernatant was used to assay PLD activity. Using 1,3-phosphatidylcholine, the substrate emulsion was prepared based on Sajdok et al*.* (1995)^46^. A standard curve for choline was derived by diluting 20 mg of choline chloride in 100 mL of acetate buffer (100 mM, pH 5.6). One unit of PLD activity was defined as mmol kg^−1^ h^−1^ choline^26^.

### Statistical analysis

The experiments were conducted as split plots for time on the basis of completely randomized design. Analysis of variance (ANOVA) was performed using SPSS software. Significant differences were calculated by Tukey’s mean test. Differences at P ≤ 0.05 were considered significant.

## Supplementary Information

Supplementary Figures.
