# Supplementary material for: Pre-harvest spray of GABA and spermine delays postharvest senescence and alleviates chilling injury of gerbera cut flowers during cold storage
Source: Sci Rep. 2021 Jul 8;11:14166. doi: 10.1038/s41598-021-93377-4 (PMC8266912; doi:10.1038/s41598-021-93377-4)
Supplement: Supplementary file 1 — Supplementary Figures. [file 41598_2021_93377_MOESM1_ESM.pdf]

Pre-harvest spray of GABA and spermine delays postharvest senescence and alleviates chilling injury of gerbera cut flowers during cold storage

Meisam Mohammadi[1,2]**, Mitra Aelaei**[1*]**, Mehdi Saidi**[2]

[1] Department of Horticulture, Faculty of Agriculture, University of Zanjan, Zanjan 45371-38791, Iran

[2] Department of Horticulture, Faculty of Agriculture, Ilam University, Ilam 69315-516, Iran

[*]**Corresponding authors**: Maelaei@znu.ac.ir

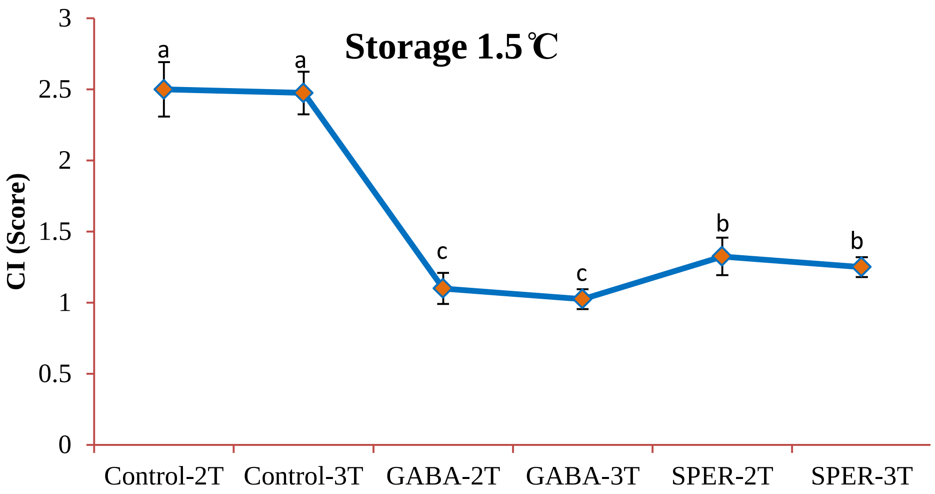

Supplementary Fig. S1. Chilling injury (CI) index of gerbera 'Stanza' cut flowers pre-treated with GABA and spermine (SPER). Means with the similar letter in each row are not significantly different at the $P < 0.05$ level of Tukey test. Standard errors (error bars) of four independent biological replicates ($n = 4$) each replicate included 36 cut flowers. 2T and 3T represent the number of pre-harvest GABA and SPER spraying on gerbera plants.

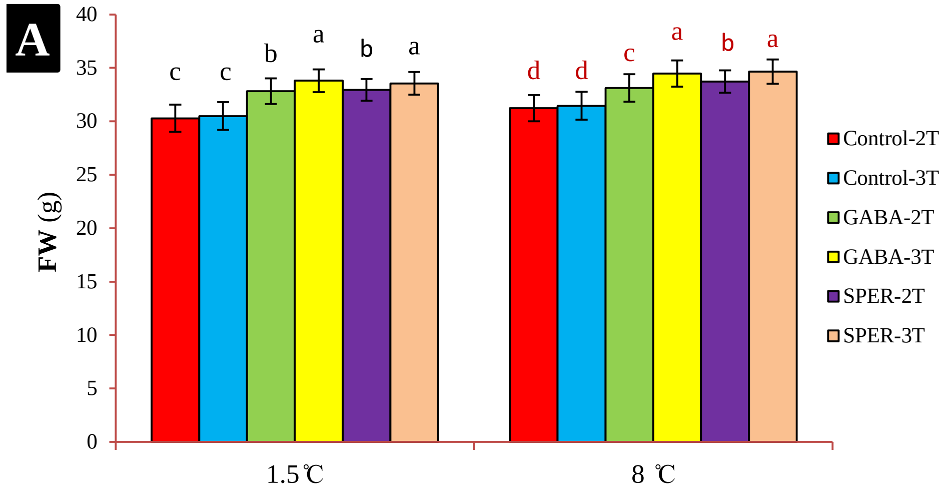

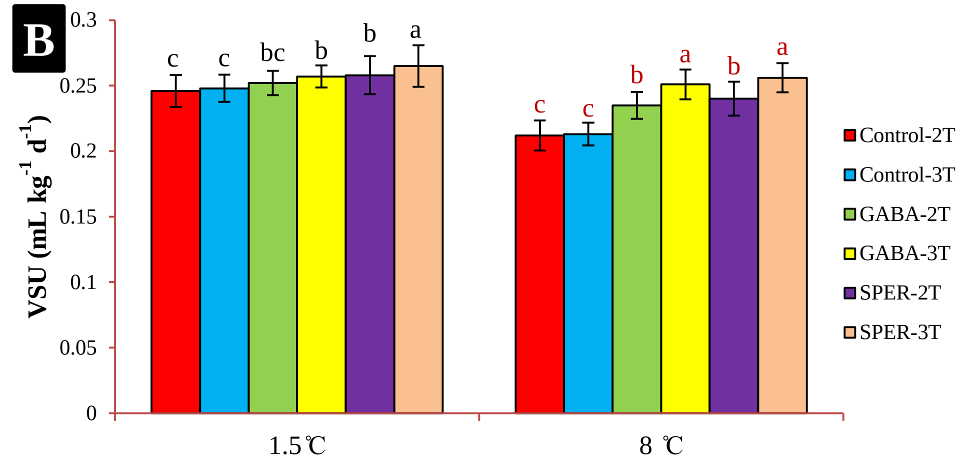

Supplementary Fig. S2. Effect of GABA and SPER pre-treatments on (**A**) fresh weight (FW) and (**B**) vase solution uptake (VSU) of gerbera 'Stanza' cut flowers during cold storage. Means with the similar letter in each row are not significantly different at the $P < 0.05$ level of Tukey test. Standard errors (error bars) of four independent biological replicates (n=4) each replicate included 36 cut flowers. 2T and 3T represent the number of pre-harvest GABA and SPER spraying on gerbera plants.

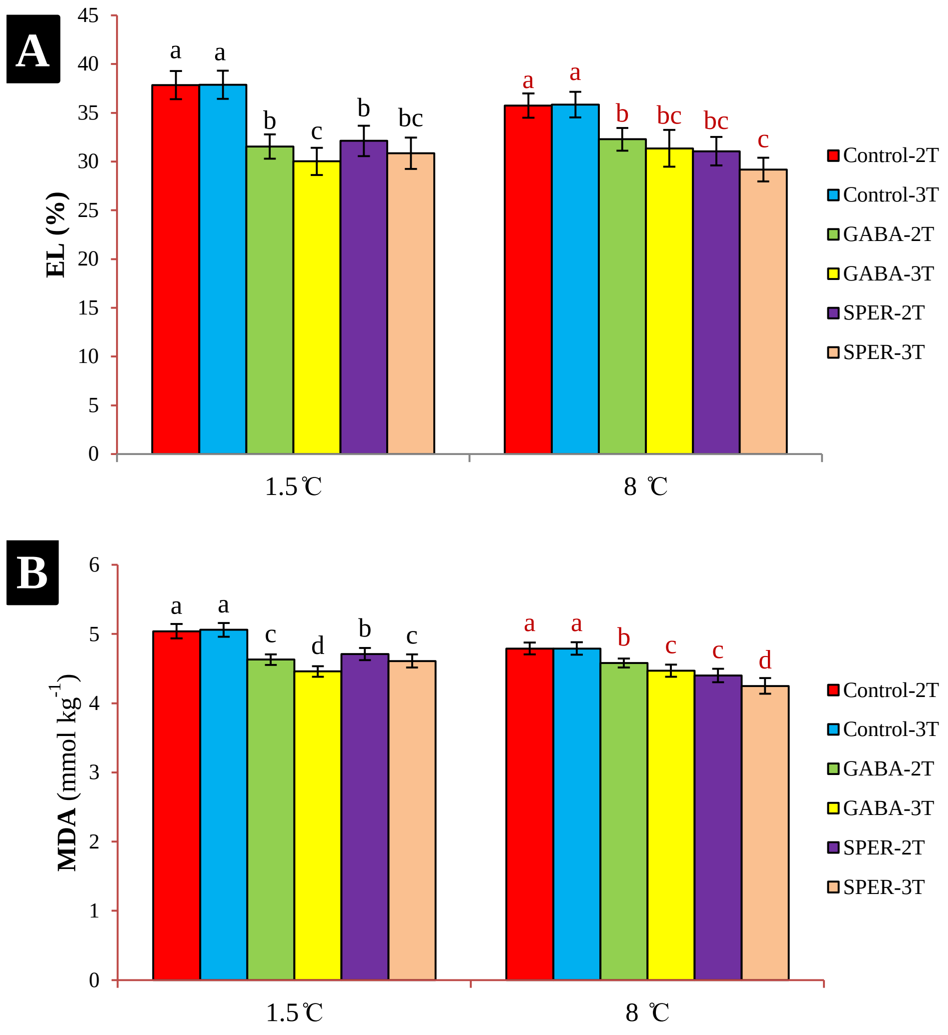

Supplementary Fig. S3. Effect of GABA and SPER pre-treatments on (**A**) electrolyte leakage (EL) and (**B**) malondialdehyde (MDA) contents of gerbera 'Stanza' cut flowers during cold storage. Means with the similar letter in each row are not significantly different at the $P < 0.05$ level of Tukey test. Standard errors (error bars) of four independent biological replicates ($n = 4$) each replicate included 36 cut flowers. 2T and 3T represent the number of pre-harvest GABA and SPER spraying on gerbera plants.

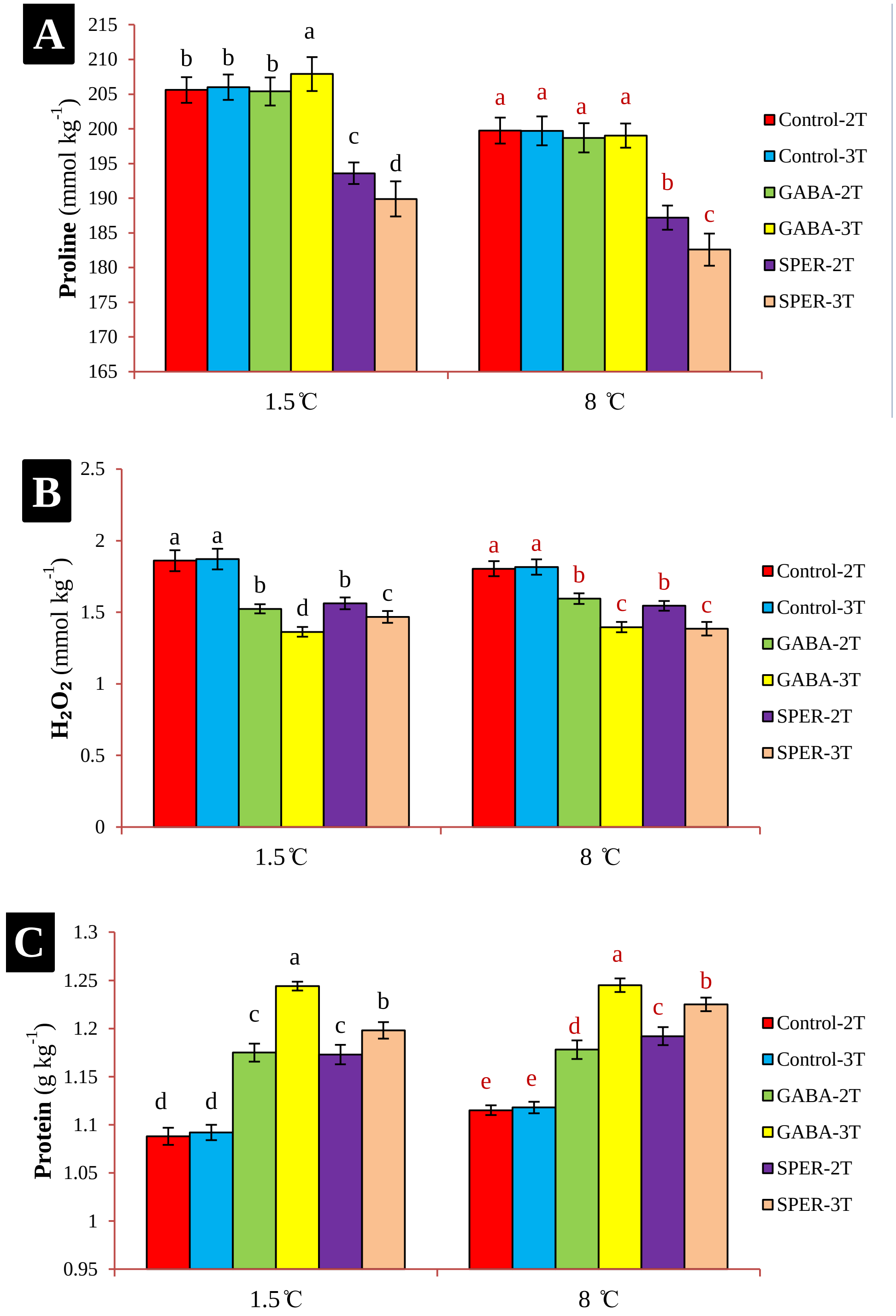

Supplementary Fig. S4. Effect of GABA and SPER pre-treatments on (**A**) proline, (**B**) hydrogen peroxide (H_2O_2) and **(C)** total protein contents of gerbera 'Stanza' cut flowers during cold storage. Means with the similar letter in each row are not significantly different at the $P < 0.05$ level of Tukey test. Standard errors (error bars) of four independent biological replicates ($n = 4$) each replicate included 36 cut flowers. 2T and 3T represent the number of pre-harvest GABA and SPER spraying on gerbera plants.

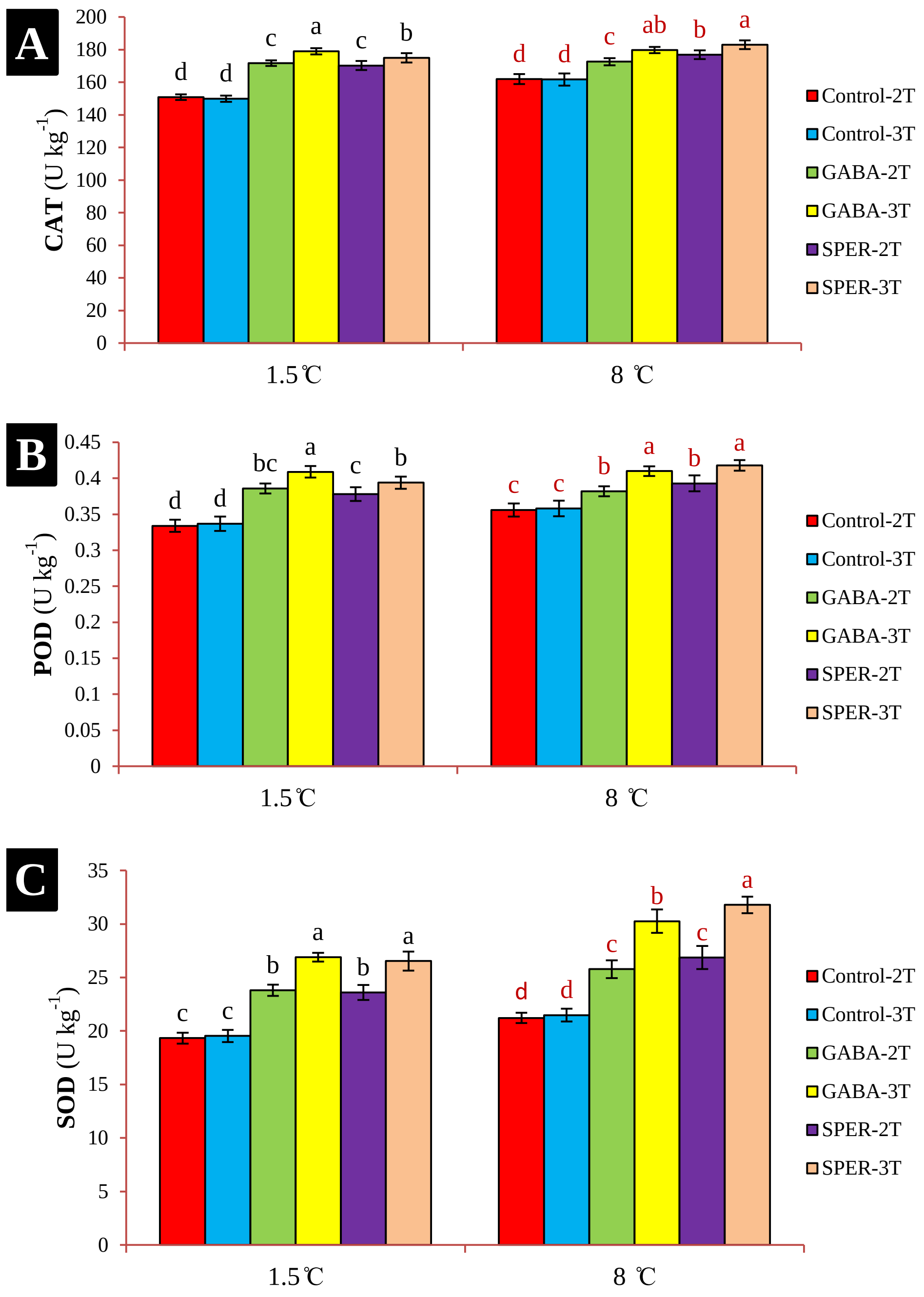

Supplementary Fig. S5. Effect of GABA and SPER pre-treatments on (**A**) catalase (CAT), (**B**) peroxidase (POD) and (**C**) superoxide dismutase (SOD) activities of gerbera 'Stanza' cut flowers during storage. Means with the similar letter in each row are not significantly different at the $P < 0.05$ level of Tukey test. Standard errors (error bars) of four independent biological replicates ($n = 4$) each replicate included 36 cut flowers. 2T and 3T represent the number of pre-harvest GABA and SPER spraying on gerbera plants.

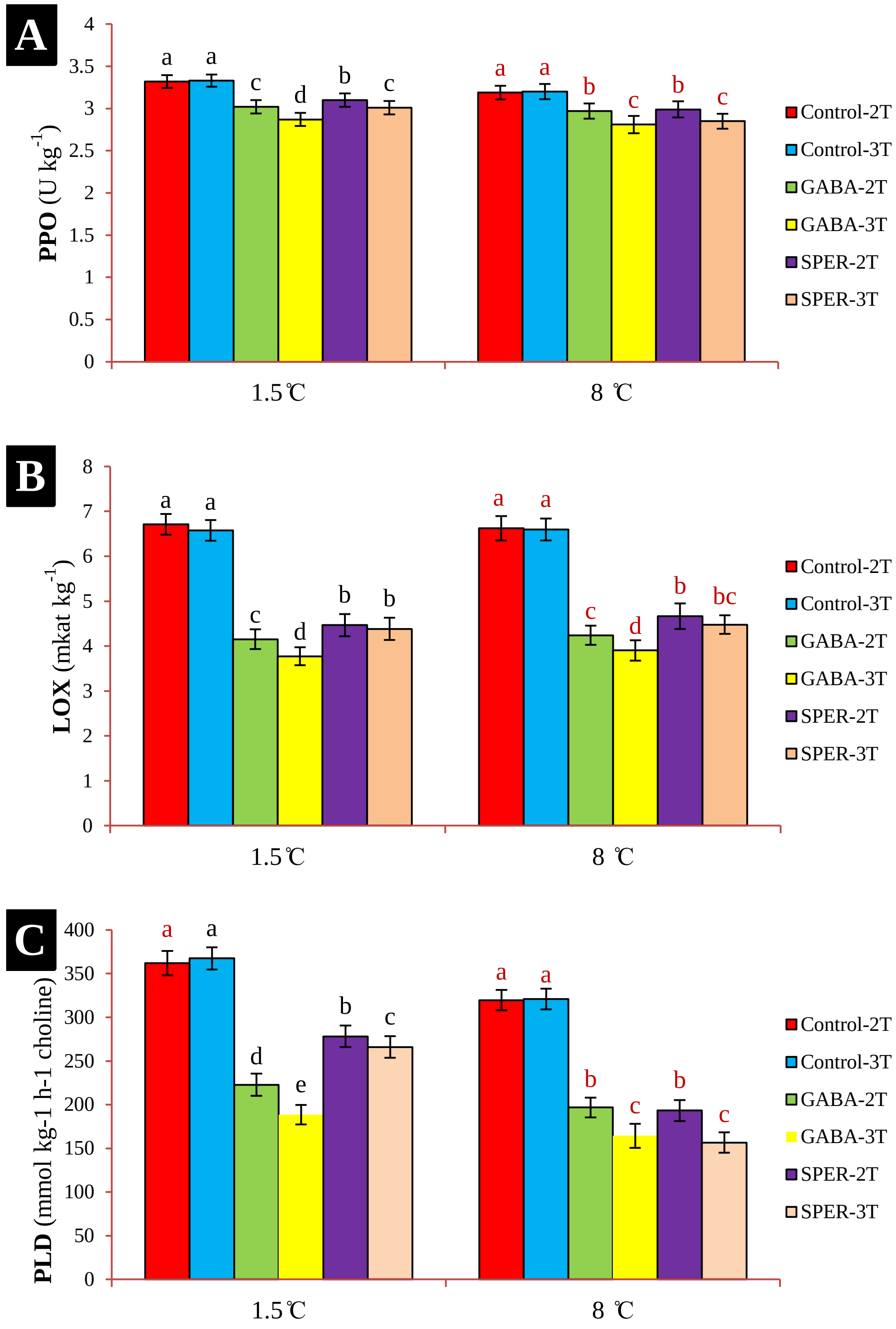

Supplementary Fig. S6. Effect of GABA and SPER pre-treatments on **(A)** polyphenol oxidase (PPO), (**B**) lipoxygenase (LOX) and (**C**) phospholipase D (PLD) activities of gerbera 'Stanza' cut flowers during storage. Means with the similar letter in each row are not significantly different at the P < 0.05 level of Tukey test. Standard errors (error bars) of four independent biological replicates (n = 4) each replicate included 36 cut flowers. 2T and 3T represent the number of pre-harvest GABA and SPER spraying on gerbera plants.

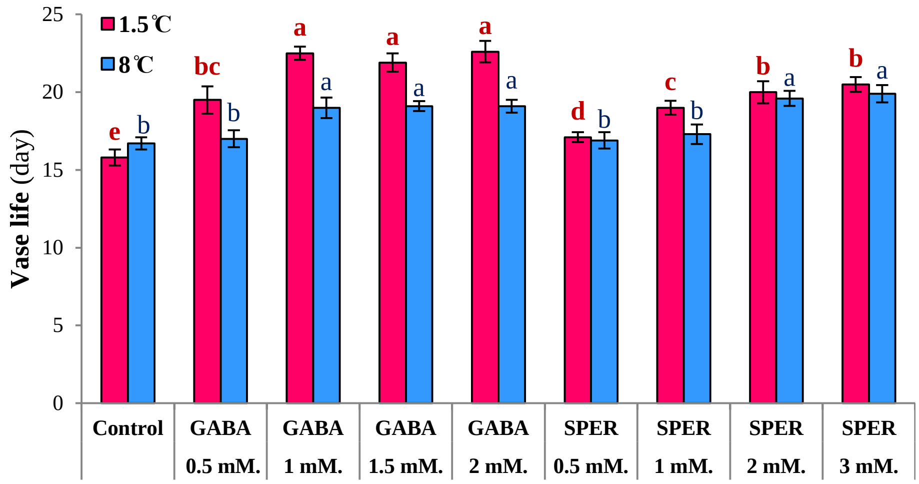

Supplementary Fig. S7. Vase life of gerbera 'Stanza' cut flowers pre-treated with different concentrations of GABA and SPER under cold storage. Means with the similar letter (s) in each row are not significantly different at the $P < 0.05$ level of Tukey test. Standard errors (error bars) of four independent biological replicates (n=4) each included 36 cut flowers.

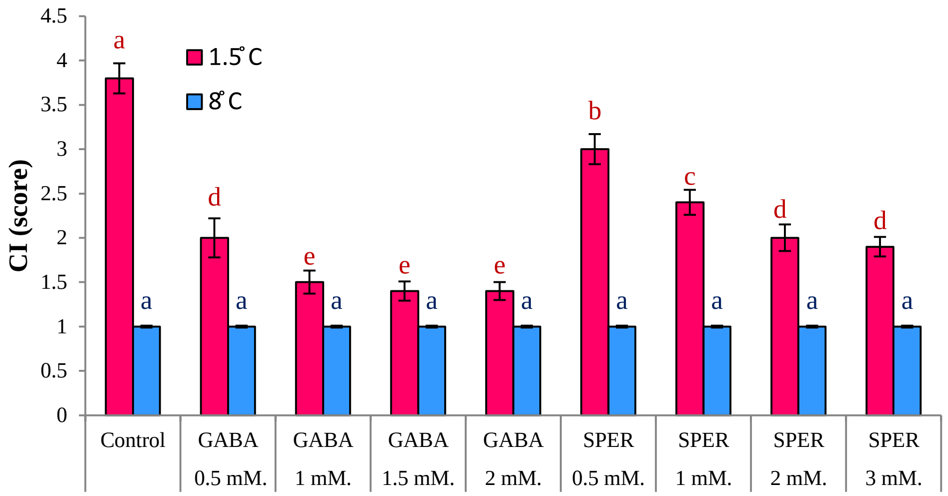

Supplementary Fig. S8. Chilling injury (CI) index of gerbera 'Stanza' cut flowers pre-treated with different concentrations of GABA and SPER at 15 days postharvest (dph). Means with the similar letter (s) in each row are not significantly different at the $P < 0.05$ level of Tukey test. Standard errors (error bars) of four independent biological replicates (n=4) each included 36 cut flowers.

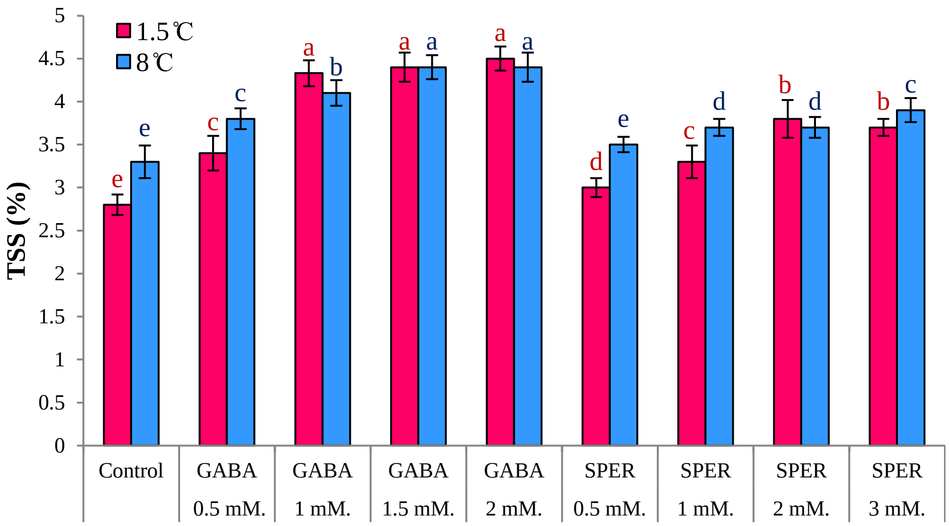

Supplementary Fig. S9. Total soluble solids (TSS) of gerbera 'Stanza' cut flowers pre-treated with different concentrations of GABA and SPER at 15 dph. Means with the similar letter (s) in each row are not significantly different at the $P < 0.05$ level of Tukey test. Standard errors (error bars) of four independent biological replicates (n=4) each included 36 cut flowers.
